# Urinary tract infection by a rare pathogen *Cedecea neteri* in a pregnant female with Polyhydramnios: rare case report from UAE

**DOI:** 10.1186/s12879-021-06298-y

**Published:** 2021-07-02

**Authors:** Hafiz Ahmad, Talat Masroor, Satyam A. Parmar, Debadatta Panigrahi

**Affiliations:** 1 Department of Medical Microbiology & Immunology, RAK College of Medical Sciences, RAK Medical and Health Sciences University, Ras al Khaimah, United Arab Emirates; 2 Clinical Laboratory, RAK hospital, Ras al Khaimah, UAE; 3 Department of Obstetrics & Gynecology, RAK Hospital, Ras al Khaimah, UAE; 4 College of Medicine, Ajman University, Ajman, UAE

**Keywords:** *Cedecea neteri*, Rare pathogen, Urinary tract infection, Multi-drug resistance

## Abstract

**Background:**

*Cedecea neteri* is a gram-negative, oxidase-negative bacillus, a rare pathogen. Few reports are emerging globally about its antimicrobial resistance pattern especially in immunocompromised individuals with comorbidities.

**Case presentation:**

In this paper, we report the first case of *C. neteri* causing urinary tract infection in a pregnant woman at a specialty care hospital in the Northern Emirates of Ras al Khaimah, UAE.

**Discussion and conclusion:**

*C. neteri* is a rare and unusual pathogen, unlike routine gram-negative urinary tract pathogens from the family of *Enterobacteriaceae* and therefore may be missed or misidentified by routine laboratories using conventional microbiology identification techniques. Hence, *Cedecea* infections may be under-reported. Physicians and microbiology technicians must be aware of such a rare pathogen, as most of the isolates are multi-drug-resistant and require combined antibiotic treatment with beta-lactamase inhibitors and hence pose a treatment challenge especially in immunocompromised patients with comorbidities. In recent years, it has been reported as an emerging opportunistic pathogen.

## Background

*Cedecea neteri* is a gram-negative, oxidase-negative bacillus – a rare pathogen with increasing case reports describing infections by the genus *Cedecea*. However, there have been very few cases of *C. neteri* reported so far, and none from urinary tract infection (UTI). In this paper, we report the first case to the best of our knowledge of *C. neteri* from a 27-year-old, young pregnant female with 35 weeks’ gestation presenting with polyhydramnios.

*Cedecea* constitutes a rare pathogen of increasing importance. *Cedecea* species are known to have antibiotic resistance genes and therefore difficult to treat infections caused by them, due to their broad-spectrum antibiotic resistance. There is emerging literature with case reports of bacteremia caused by *C. neteri* and more cases reported by other *Cedecea* species: *C. davisae* and *C.lapagei*. Nevertheless, the classical case of UTI by *C. neteri* species is rare. Literature review showing case of urinary catheter colonization by multidrug-resistant *C. neteri* in an elderly 88-year-old immunocompromised patient presenting with cellulitis, benign prostatic hyperplasia, and prolong use of Foleys catheter. The *C.neteri* isolated from urinary catheter was sensitive to most 2nd and 3rd generation cephalosporins and resistant to ampicillin along with *β*-lactamase inhibitor (Sulbactam) combination, suggesting AmpC *β*-lactamase production with the presence of multiple metallo *β*-lactamase genes [1]. In another report from Turkey, described UTI caused by *Cedecea lapagei* in a 40-year male with invasive brain surgery after 3 weeks of rehabilitation and was successfully treated with Ciprofloxacin [2]. Furthermore, there have been emerging reports of the association of *C. neteri* with other clinical presentations. To mention a few, a report of bacteremia in patients of heart disease [3], in patients with systemic lupus erythematosus [4], and a case report from Saudi Arabia where C*. neteri* was isolated from peritoneal fluid of an immuno-compromised preterm neonate following intestinal perforation due to necrotizing enterocolitis [5]. Infections from *Cedecea* are mostly reported from immunocompromised patients with multiple comorbidities or invasive procedures, suggesting its role as an emerging opportunistic multi-drug resistant pathogen [1, 3, 5].

## Case presentation

Our patient was a 27-year-old pregnant woman at 35 weeks’ gestation who visited the Gynecology outpatient department of RAK hospital with complaints of increased frequency, urgency, and dysuria; classical presentation of UTI. The patient was on follow-up for polyhydramnios. On examination, she was afebrile, well oriented to time, place, and person, with a pulse rate of 86/min, blood pressure of 152/90 mmHg, a known hypertensive. However, there were no signs of cystitis or pyelonephritis based on abdominal and pelvis ultrasound. Her history revealed that she was multiparous and her first pregnancy ended with twins born by caesarian. During the second pregnancy, she developed polyhydramnios and was on continuous follow-up. Her mid-stream first-morning urine sample macroscopically was cloudy with yellow coloration. Microscopically it showed significant polymorph nuclear cells (18–20 cells/high power field), no RBCs, and 3+ leukocyte esterase activity with plenty of leukocytes and bacteria with < 1 squamous epithelial cell. Culture and identification yielded significant bacteriuria of 10^5^ CFU/ml of a gram-negative bacterium grown in Cysteine Lactose Electrolyte Deficient (CLED) agar. On further characterization, the isolate was identified by Phoenix automated microbial identification system (BD Diagnostics, Spars, Maryland), belonging to genus *Cedecea* and species *neteri.* To confirm the diagnosis of UTI, two mid-stream urine samples on consecutive days were further cultured. Both samples showed significant growth (10^5^ CFU/ml) of *C. neteri* and thus confirmed the diagnosis.

Antimicrobial susceptibility testing showed the isolate to be resistant to Amikacin, Aztreonam, Cephalothin, Ceftriaxone, Ertapenem, Cefepime, Cefoxitin and susceptible to Ampicillin, Ampicloxacillin, Nitrofurantoin, Levofloxacin, Tigecycline & Piperacillin/Tazobactum. The patient was treated with one gram of Augmentin (combination of 875 mg amoxicillin and 125 mg clavulanic acid) for 5 days and urine culture was sterile post 1 week of treatment.

## Discussion and conclusion

In females, UTI is most often caused by members of the family *Enterobacteriaceae* mainly *E. coli,* mostly endogenous, due to the proximity of the anus to the urethra and short urethra causing ascending infections in women. To the best of our knowledge, this is the first case of a UTI by a rare pathogen, *Cedecea neteri,* reported from the UAE, in a 35-week pregnant, multiparous female who has polyhydramnios and a known case of hypertension. Most likely *C. neteri* has been a colonic microbial flora of this patient.

*Cedecea* was described in 1981 as a separate genus of the *Enterobacteriaceae* family as it was phenotypically distinct from the other members of the family and named after CDC, where the isolates were originally discovered and formerly called as CDC Enteric group 15 [6]. To date, six species of *Cedecea* have been reported with three species, *Cedecea neteri*, *Cedecea lapagei*, and *Cedecea davisae*, that are well characterized. *C. neteri* is named after the American physician-microbiologist Dr. Erwin Neter [7]. *Cedecea* species have not been reported to cause invasive infection in healthy individuals, but are considered opportunistic pathogens due to their isolation from severely immunocompromised patients. The case presented here is a pregnant mother who is immunocompromised and therefore more susceptible to infections. The growing fetus also exerts pressure on the urinary bladder, increasing residual urine, thereby affecting complete voiding and urine stasis predisposing to UTI.

These pathogens may be missed or misidentified by routine laboratories using conventional microbiology identification methods and hence may be under-reported. *Cedecea neteri* strains are gram-negative, rod-shaped, motile [8]. They are characterized by positive biochemical reactions for sucrose, d-sorbitol, and malonate fermentation tests [3, 9]. Clinical history, laboratory diagnosis, and antimicrobial resistance pattern of *Cedecea* spp. resemblance to established pathogen *Serratia* spp. in terms of lipase positivity and resistance to cephalothin, and colistin (polymyxin E) [3, 7, 8]. *C. neteri* is distinguished from other *Cedecea* spp. with negative ornithine decarboxylase activity and its ability to ferment sucrose, D-sorbitol, and D-xylose [3].

Multidrug resistance in *Cedecea* spp. is attributed to the combination of AmpC production and porin deficiency in the cell wall [8, 10]. However, no definite experimental evidence on this theory is available to this day. *Cedecea* clinical isolates display variable resistance patterns to major beta-lactam like penicillins, cephalosporins, monobactam, and carbapenems. The latest literature review indicates the highest frequency of *Cedecea* spp. resistance to ampicillin (43%), followed by cephalothin (35%), cefoxitin (35%), cefazolin (22%), ceftazidime from a total of 23 isolates reported to date [11]. Carbapenems and 4th generation cephalosporin resistance may be exhibited in *Cedecea* isolates harboring metallo-*β*-lactamase. *C. neteri* poses another unique feature to enhance its pathogenicity by Quorum sensing activity that has been lately studied in *C. neteri* strain SSMF04 and can lead to enhanced biofilm formation as well as enhanced antimicrobial resistance [12]. Fortunately, low resistance to fluoroquinolones and no resistance to macrolides have been documented. It is also encouraging to note that most of the reported cases have successful treatment outcomes and an active antibiotic stewardship policy with proactive microbiology laboratory diagnosis and antibiotic sensitivity testing can improve clinical outcomes. *C. neteri* infections have been reported worldwide, with clinical cases occurring from U.S., Spain, Saudi Arabia, and the latest one from UAE (Table 1. summarizes all the *C. neteri* cases reported to date).

**Table 1:** Cases of *Cedecae neteri* reported to date

Study year	Patient (age/sex, location)	***Cedecea*** spp.	Infection	Diagnosis	Antibiotic Sensitivity/Treatment	Antibiotic Resistance	Clinical Outcome	Reference
2021	27/F UAE	*C. neteri*	Urinary tract Infection	Polyhydramnios, hypertension	amoxicillin and clavulanic acid	Amikacin, Aztreonam, Cephalothin, Ceftriaxone, Ertapenem, Cefepime, Cefoxitin.	Successful recovery	Hafiz et al. 2021
2018	88/M USA	*C. neteri*	Colonization of the Urinary catheter	Cellulitis, with hypertension, chronic kidney disease and benign prostatic hyperplasia	cefmandole, ceftazidime, ceftriaxone, cefepime, aztreonam, nitrofurantoin, ciprofloxacin, TMP/SMX Piperacillin/tazobactam,	Ampicillin/sulbactam, cefazolin, cefoxitin	Successful recovery	Ginn et al. [1]
2017	Neonate/M Saudi Arabia	*C. neteri*	Peritonitis	Perforation of Intestine	Not reported	Piperacillin/tazobactam and gentamicin	Successful recovery	Arishi et al. [5]
1995	27/F Spain	*C. neteri*	Bacteremia	systemic lupus erthematosus	Intravenous vancomycin, ceftazidime, gentamicin	Amoxicillin, cephalosporins, amoxicillin and clavulanic acid, aminoglycosides	Died	Augileria et al. [4]
1982	62/M USA	*C. neteri*	Bacteremia	Cardiac and valvular heart disease	Cefamandole, chloramphenicol, n	Cefalothin, ampicillin, colistin	Successful recovery	Farmer et al. [3]

As summarized in Table 2, most *Cedecea* infections are reported in immunocompromised patients with underlying medical conditions like uncontrolled diabetes mellitus, chronic kidney disease, liver transplantation, malignancies, chronic obstructive pulmonary disease, and few isolated cases from catheters and central lines, indicating its opportunistic potential and warranting careful attention among these groups of patients.

**Table 2:** Summary of representative *Cedecea spp.* cases reported to date

Study year	Patient (age /sex, location)	***Cedecea*** spp.	Infection	Diagnosis	Antibiotic Resistance	Treatment	Clinical Outcome	Reference
2019	Neonate/India	*C. lapagei*	Nosocomial Pneumonia	Late preterm	Meropenem, Colisitin, amikacin, ceftazidime	Piperacillin/tazobactam	Recovered	Ramaswamy et al. 2019 [13]
2019	41/F USA	*C. davisae*	Biliary sepsis	Minimal change disease	Ampicillin, ceftriaxone, cefuroxime	Ciprofloxacin, metronidazole	Recovered	Kanakadandi et al. [10]
2018	52/M Mexico	*C. lapagei*	Soft tissue bullae leading to septic shock	Liver cirrhosis, esophageal varices, hypertension	Ampicillin, cefazolin, imipenem; ampicillin/sulbactam	Intravenous imipenem, clindamycin	Died	Chavez Herrera et al. 2018 [14]
2017	Neonate/M Brazil	*C. lapagei*	Ventilator-associated pneumonia, sepsis		Not reported	Multiple courses of antibiotics including meropenem	Recovered	Kury et al. [15]
2017	Neonate/M India	*C. lapagei*	Late-onset sepsis	Preterm	Imipenem, meropenem, aztreonam, ceftazidime, cefotaxime, cefoxitin, cefepime,	Ampicillin, cefotaxime	Recovered	Ahmad et al. [16]
2016	Neonate/F India	*C. lapagei*	Neonatal sepsis	Term infant	Amoxicillin/clavulanic acid, ceftazidime, ceftriaxone, gentamicin, cefuroxime, piperacillin/tazobactam, TMP/SMX	Ciprofloxacin, amikacin	Recovered	Islam et al. [17]
2015	50/M India	*C. lapagei*	Superinfection of malignant oral ulcer	Squamous cell carcinoma of the right buccal mucosa	Ampicillin/sulbactam,tetracycline, tigecycline	Ciprofloxacin,	Recovered	Biswal et al. [18]
2006	55/M USA	*C. lapagei*	CAPD-related peritonitis	CAPD, Hypertension, Liver transplantation, cirrhosis, end-stage renal disease	Not reported	Initially intravenous vancomycin & gentamicin, followed by ceftazidime & gentamicin added to PD	Recovered	Davis & wall [19]
2012	54/M Greece	*C. davisae*	Bacteremia	Stage IV sigmoid colon carcinoma	Tobramycin	Gentamicin	Recovered	Akinosoglou et al. [20]
2012	20/M USA	*C. davisae*	Polymicrobial* HCA pneumonia	Cystic Fibrosis	All tested β-lactams, aminoglycosides, fluoroquinolones, tigecycline	TMP/SMX	Recovered	Ismael et al. [21]
2011	52.M USA	*C. davisae*	Central line-related bacteremia	Acute myeloid leukemia, neutropenia, *C. difficile* colitis	Ceftazidime, ciprofloxacin, piperacillin/tazobactam	Imipenem	Recovered	Abate et al. [22]
2008	67/M Greece	*C. davisae*	Leg ulcer, bacteremeia	Uncomplicated DM	Cephalothin cefuroxime sodium, cefoxitin, ampicillin, piperacillin, nitrofurantoin, tetracycline	Cefotaxime, amikacin	Recovered	Dalamaga et al. [23]
2008	47/M Greece	*C. lapagei*	Bacteremia and wound infection from cement-related chemical burns	Diabetes mellitus	Gentamicin, Tobramycin, cephalothin, cefoxitin, sodium, cefoxitin, ampicillin, piperacillin, nitrofurantoin, tetracycline	Cefotaxime, amikacin	Recovered	Dalamaga et al. [24]
1983	50/M USA	*C. davisae*	Scrotal Abscess	Chronic hypertensive cardiac disease, alcoholic hepatitis	Ampicillin, cephalothin, cefamandole, cefoxitin, chloramphenicol	Tetracycline	Recovered	Bae & Sureka [25]

*C. davisae* infections have been reported from a broad clinical spectrum with bacteremia as a common clinical presentation. Thirteen cases have been reported to date from different clinical specimens including; 46% from blood, 23% from sputum, urine, cutaneous and oral ulcers, and scrotal abscess. It is also noteworthy that the majority of infections were among patients > 50 years of age with co-morbid conditions.

Apart from the endogenous source of infections from the gut, *Cedecea* infections are also documented from environmental samples and aquatic habitats. A case report of a patient with minor leg ulcer infection and subsequent bacteremia (Dalmaga et al. 2008) raises concerns of *C. davisae* infection from lake water.

*C. lapagei* have been more frequently isolated with pneumonia and bloodstream infections and to date, a total of 11 cases have been reported from blood, sputum, tissue, wound, peritoneal fluid and most patients had co-morbid conditions like acute leukemia, type II diabetes mellitus, pulmonary tuberculosis, liver cirrhosis, and chronic obstructive pulmonary disease. Pediatric infections involving *Cedecea* are largely associated with *C.lapagei,* suggesting its role as an uncommon cause of nosocomial pneumonia and sepsis in infants with a history of multiple antibiotic treatment regimens and prolonged hospitalization.

It is also noteworthy, that most of the *Cedecea* infections are resistant to quinolones (ciprofloxacin, Cefotaxime (third-generation cephalosporin), Imipenem, and Meropenem (carbapenems) and intravenous combination with β-lactamase inhibitors (*Piperacillin* and *Tazobactam) making it a potential multidrug-resistant pathogen.* Therefore, clinicians and diagnostic laboratories need to be aware of such rare pathogens that are often drug-resistant and require combined antimicrobial therapy. A complete antimicrobial susceptibility testing profile, especially with beta-lactam inhibitors is warranted to provide the best treatment options, especially among immunocompromised patients with comorbidities. (Tables 1, 2) The present reported *C. neteri* isolate was resistant to most of the third and fourth generation cephalosporin except a few amino and carboxypenicillins that provided a successful treatment outcome. This report further adds to the existing literature on diagnosis, clinical presentation, antibiotic susceptibility, and clinical management of *C. neteri* infection.

## Data Availability

All patient data is coded and kept confidential under authorization control in the hospital’s Laboratory Information Management System and cannot be shared. However, parts of Cedecea neteri report generated by automated microbiology system can be shared upon request without compromising patient information. The data is user restricted and was accessed by the study PI- Dr. Hafiz Ahmad who is also a clinical microbiologist at the Department of Microbiology at RAK Hospital, Ras al Khaimah, UAE.

## Abbreviations

RAK: Ras al Khaimah
UTI: Urinary Tract Infections (UTI)
CLED Agar: Cysteine Lactose Electrolyte Deficient Agar
CFU: Colony Forming Unit
CDC: Centers for Disease Control and Prevention
U.A.E: United Arab Emirates

## References

[1] Ginn PS, Tart SB, Sharkady SM, Thompson DK. Urinary Catheter Colonization by Multidrug-Resistant Cedecea neteri in Patient with Benign Prostatic Hyperplasia. Case Rep Infect Dis. 2018:1–5. 10.1155/2018/7520527. 10.1155/2018/7520527 PMC6079608 30123589

[2] Çekin Y, Kızılateş F, Dolu S, Öztoprak N, Çekin AH (2014). The first urinary tract infection caused by *Cedecea lapagei*: a case report and review of the literature. Gazi Med J.

[3] Farmer JJ, Sheth NK, Hudzinski JA, Rose HD, Asbury MF (1982). Bacteremia due to *Cedecea neteri* sp. nov. J Clin Microbiol.

[4] Aguilera A, Pascual J, Loza E, Lopez J, Garcia G, Liaño F (1995). Bacteremia with *Cedecea neteri* in a patient with systemic lupus erythematosus. Postgrad Med J.

[5] Arishi HM, Daghriri AM, Gumairy FY, Ali YF (2017). *Cedecea neteri* peritonitis as a complication of necrotizing Enterocolitis in a neonate. JCR.

[6] Janda JM, Abbott SL (2006). Uncommon enterobacterial genera associated with clinical specimens. The Enterobacteria.

[7] Dalamaga M, Sotiropoulos GP, Vrioni G, Tsakris A (2014). *Cedecea*: an "unknown" pathogen in the family of *Enterobacteriaceae* - its clinical importance, detection, and identification methods. Acta Microbiologica Hellenica [Internet].

[8] Ammenouche N, Dupont H, Mammeri H (2014). Characterization of a Novel AmpC -Lactamase Produced by a Carbapenem-Resistant *Cedecea davisae* Clinical Isolate. Antimicrob Agents Chemother.

[9] Grimont PAD, Grimont F, Farmer JJ, Asbury MA (1981). Cedecea davisae gen, nov.,ap. Nov. and Cedecae lapagei sp. Nov., new *Enterobacteriaceae* from clinical specimens. Int J Syst Bacteriol.

[10] Kanakadandi V, Sarao MS, Cunningham JM (2019). A rare case of *Cedecea davisae* bacteremia presenting as biliary sepsis. Cureus.

[11] Thompson DK, Sharkady SM (2020). Expanding Spectrum of opportunistic Cedecea infections: current clinical status and multidrug resistance. Int J Infect Dis.

[12] Tan KH, Tan JY, Yin WF, Chan KG (2015). Genome analysis of quorum sensing *Cedecea neteri* SSMD04 leads to identification of its novel signaling synthase (cneI), cognate receptor (cneR) and an orphan receptor. PeerJ.

[13] Ramaswamy VV, Gummadapu S, Suryanarayana N (2019). Nosocomial pneumonia and sepsis caused by a rare organism *Cedecea lapagei* in an infant and a review of literature. BMJ Case Rep.

[14] Chavez Herrera VR, Rosas De Silva MF, Orendain Alcaraz H, Ceja Espiritu G, Carrazco Peña K, Melnikov V (2018). Death related to *Cedecea lapagei* in a soft tissue bullae infection: a case report. J Med Case Rep.

[15] Kury CMH, Yabrudi AA, de Souza TB, de Souza EC, E Silva Costa LT, Soares CB, et al. (2017). First reported case of ventilator-associated pneumonia and sepsis caused by *Cedecea lapagei* in a Brazilian neonatal intensive care unit. J Pediatr Infect Dis Soc.

[16] Ahmad N, Ali SM, Khan AU (2017). First reported New Delhi metallo-β-lactamase-1-producing *Cedecea lapagei*. Int J Antimicrob Agents.

[17] Islam AKS, Bora R, Ahmed R, Borah AK, Ramasamy S (2016). A case of neonatal sepsis with pneumonia due to *Cedecea lapagei*. IOSR-JDMS.

[18] Biswal I, Hussain NA, Grover RK (2015). *Cedecea lapagei* in a patient with malignancy: report of a rare case. J Cancer Res Ther.

[19] Davis O, Wall BM (2006). “Broom straw peritonitis” secondary to *Cedecea lapagei* in a liver transplant recipient. Perit Dial Int.

[20] Akinosoglou K, Perperis A, Siagris D, Goutou P, Spiliopoulou I, Gogos CA, Marangos M (2012). Bacteraemia due to *Cedecea davisae* in a patient with sigmoid colon cancer: a case report and brief review of the literature. Diagn Microbiol Infect Dis.

[21] Ismaael TG, Zamora EM, Khasawneh FA (2012). *Cedecea davisae*’s role in a polymicrobial lung infection in a cystic fibrosis patient. Case Rep Infect Dis.

[22] Abate G, Qureshi S, Mazumder SA (2011). *Cedecea davisae* bacteremia in a neutropenic patient with acute myeloid leukemia. J Inf Secur.

[23] Dalamaga M, Karmaniolas K, Arsenis G, Pantelaki M, Daskalopoulou K, Papadavid E, Migdalis I (2008). *Cedecea lapagei* bacteremia following cement-related chemical burn injury. Burns.

[24] Dalamaga M, Pantelaki M, Karmaniolas K, Matekovits A, Daskalopoulou K (2008). Leg ulcer and bacteremia due to *Cedecea davisae*. Eur J Dermatol.

[25] Bae BHC, Sureka SB (1983). *Cedecea davisae* isolated from scrotal abscess. J Urol.

